# Effectiveness and safety of misoprostol distributed to antenatal women to prevent postpartum haemorrhage after child-births: a stepped-wedge cluster-randomized trial

**DOI:** 10.1186/s12884-015-0750-6

**Published:** 2015-11-26

**Authors:** Sam Ononge, Oona M. R. Campbell, Frank Kaharuza, James J. Lewis, Katherine Fielding, Florence Mirembe

**Affiliations:** Department of Obstetrics and Gynaecology, Makerere University College of Health Sciences, PO Box 7072, Kampala, Uganda; Department of Epidemiology and Population Health, London School of Hygiene and Tropical Medicine, Keppel Street WC1E 7HT, London, UK; School of Public Health, Makerere University College of Health Sciences, PO Box 7072 Kampala, Uganda; MRC Tropical Epidemiology Group, London School of Hygiene and Tropical Medicine, Keppel Street WC1E 7HT, London, UK

**Keywords:** Acceptability, Antenatal distribution, Home births, Misoprostol, Postpartum haemorrhage, Safety, Stepped-wedge cluster trial

## Abstract

**Background:**

Oral misoprostol, administered by trained health-workers is effective and safe for preventing postpartum haemorrhage (PPH). There is interest in expanding administration of misoprostol by non-health workers, including task-shifting to pregnant women themselves. However, the use of misoprostol for preventing PPH in home-births remains controversial, due to the limited evidence to support self-administration or leaving it in the hands of non-health workers. This study aimed to determine if antenatally distributing misoprostol to pregnant women to self-administer at home birth reduces PPH.

**Methods:**

Between February 2013 and March 2014, we conducted a stepped-wedge cluster-randomized trial in six health facilities in Central Uganda. Women at 28+ weeks of gestation attending antenatal care were eligible. Women in the control-arm received the standard-of-care; while the intervention-arm were offered 600mcg of misoprostol to swallow immediately after birth of baby, when oxytocin was not available. The primary outcome (PPH) was a drop in postpartum maternal haemoglobin (Hb) by ≥ 2g/dl, lower than the prenatal Hb. Analysis was by intention-to-treat at the cluster level and we used a paired t-tests to assess whether the mean difference between the control and intervention groups was statistically significant.

**Results:**

97 % (2466/2545) of eligible women consented to participate; 1430 and 1036 in the control and intervention arms respectively. Two thousand fifty-seven of the participants were successfully followed up and 271 (13.2 %) delivered outside a health facility. There was no significant difference between the study group in number of women who received a uterotonic at birth (control 80.4 % vs intervention 91.4 %, mean difference = -11.0 %, 95 % confidence interval [CI] -25.7 % to 3.6 %, *p =* 0.11). No woman took misoprostol before their baby’s birth. Shivering and fever were 14.9 % in the control arm compared to 22.2 % in the intervention arm (mean difference = -7.2 %, 95 % CI -11.1 % to -3.7 %), *p =* 0.005). There was a slight, but non-significant, reduction in the percentage of women with Hb drop ≥ 2g/dl from 18.5% in the control arm to 11.4 % in the intervention arm (mean difference = 7.1 %, 95 % CI -3.1 % to 17.3 %, *p =* 0.14). Similarly, there was no significant difference between the groups in the primary outcome in the women who delivered at home (control 9.6 % vs intervention 14.5 %, mean difference -4.9; 95 % CI -12.7 to 2.9), *p =* 0.17).

**Conclusion:**

This study was unable to detect a significant reduction in PPH following the antenatal distribution of misoprostol.

The study was registered with Pan-African Clinical Trials Network (PACTR201303000459148, on 19/11/2012).

**Electronic supplementary material:**

The online version of this article (doi:10.1186/s12884-015-0750-6) contains supplementary material, which is available to authorized users.

## Background

Globally, maternal deaths have declined by 45 % from an estimated 543,000 maternal deaths in 1990, to 289,000 in 2013 [1]. However, this observed progress is stalled in sub-Saharan Africa, which now contributes 62 % of all maternal deaths [1]. Haemorrhage is the leading cause of maternal death in sub-Saharan Africa, accounting for an estimated 25 % of deaths [2], and postpartum haemorrhage (PPH) defined as bleeding after childbirth of 500 mls or more contributes two-thirds of these [2]. PPH can be prevented by using a uterotonic immediately after the birth of the baby, and this intervention is recommended for all women [3]. The preferred uterotonic is oxytocin [4], which is available in injectable form and requires refrigeration, making it impractical in settings where births still occur at home under the care of unskilled birth attendants, or where refrigeration is not possible. Misoprostol, a prostaglandin E_1_ analogue that induces strong uterine contractions, is an alternative [5, 6] that is cheap, heat stable, and has a long shelf-life. While it is not as effective as oxytocin [7], there is health facility and community evidence to recommend health workers giving 600 micrograms of misoprostol orally or sublingually after birth of the baby, but before delivery of the placenta, to prevent PPH, when oxytocin is not available [8–12]. There is also increasing evidence to support the safety of community distribution of misoprostol through traditional birth attendants and community health workers, which is a low-cost strategy [13–17]. In view of the emerging evidence, the World Health Organization (WHO) PPH prevention guidelines recommend using lay community health workers to administer misoprostol for PPH prevention when oxytocin is not available [4]. However, because of the low quality of evidence on the effectiveness of self-administered misoprostol use in home births [18], the WHO and maternal health experts did not recommend antenatal distribution of misoprostol to women to self-administer at birth. Rather they recommended more research at the community-level to investigate the effect of antenatal distribution of misoprostol to pregnant women to self-administer in third stage of labour in settings or situations where oxytocin use is not feasible [4, 19, 20].

Uganda is among countries categorized by WHO as ‘not on track’ in achieving MDG 5, [1] with a maternal mortality ratio estimated at 438 per 100,000 live births. PPH is the leading cause of maternal mortality and is responsible for 25 % of deaths [21]. Misoprostol was approved for preventing and treating PPH in Uganda in 2008 administered by health workers. While most (95 %) women in Uganda receive antenatal care once and 47.6 % having at least four visits, 42 % still deliver at home [21]. Giving women misoprostol antenatally to self-administer after home birth would be a good strategy to prevent PPH. However such a strategy is not yet approved in Uganda. The main concern for policy makers is whether antenatal distribution of misoprostol would encourage home delivery, at a time when the Ministry of Health is promoting health-facility delivery. The aim of the present study was to assess the effectiveness and safety of antenatal distribution of misoprostol to women to self-administer in home births in preventing PPH.

## Methods

### Ethical considerations

Ethical approval was obtained from the School of Medicine Research and Ethics Committee at Makerere University, Kampala, Uganda, and the Uganda National Council for Science and Technology. Permission to carry out the study was obtained from the District Health Office (DHO) and respective in-charges of the health facilities. After information and counselling, eligible women provided written informed consent and received an information sheet in either English or Luganda. The study was registered with the Pan African Clinical Trials Network (PACTR201303000459148) on 19/11/2012.

### Study design and randomization

**Fig. 1:**
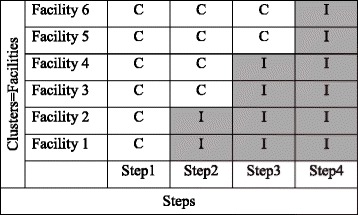
Stepped-wedge schema for the trial. Six clusters were enrolled at baseline. The white (non-shaded) cells marked “C” represent the control period. The gray (shaded) cells marked “I” represent the intervention period

We employed a stepped-wedge cluster-randomized trial design [22] because current evidence on misoprostol use and postpartum haemorrhage would render a placebo-controlled trial unethical [19, 22–25] and all facilities ultimately get the intervention. A cluster was defined as a health facility catchment area. All health facilities started as control-arm facilities. Then in a prior-determined random order, two facilities “crossed over” to become intervention facilities during each of the subsequent three steps for a total of four steps (Fig. 1).

The random sequence for starting the intervention was determined before the start of the study by using computer generated number sequence. The principal investigator implemented the randomization. Each step lasted for two months, and women were followed up on 3^rd^ to 5^th^ day post delivered. Because of the nature of the intervention, it was not possible to blind the intervention to the care-givers, research team or study participants.

### Participants and setting

The study participants were recruited from six health facilities in Mpigi district, Uganda, between February 2013 and March 2014. The majority of people in the district are of low socioeconomic status, with peasant farming and fishing as their main economic activities. The district health infrastructure consisted of 31 health units (25 government and 6 non-government). These included one private hospital, one Health Centre IV, 13 Health Centre IIIs, and 16 Health Centre IIs. The district recorded a skilled birth attendant rate of 30 % (2010–11 District Annual Report), although the national average was 58 % [21]. We enrolled study participants at the antenatal clinic of the Health Centre IV and the five Health Centre IIIs.

Two health facilities held their antenatal clinics from Monday to Friday, while the other four had two dedicated antenatal-care service days per week. Maternity services at the Health Centre IIIs were staffed by midwives, while the Health Centre IV had three medical officers in addition to the 7 midwives, and provided comprehensive emergency obstetric care. The staff in the antenatal clinic and delivery wards were involved in recruiting and following up the women, which allowed the intervention to be delivered as part of ongoing maternity care.

### Eligibility criteria for clinics and women

Clinics: All the 31 health facilities were screened for eligibility. The eligibility criteria were a) that a minimum of 50 pregnant women attended antenatal clinic (for the first time) in the month prior to the start of the study, and b) that the person in-charge of health facility agreed for the facility to participate. Of the nine facilities that registered a minimum of 50 antenatal first-time attendees per month, we excluded three; the hospital because health-care services are paid for (private hospital); one health facility because its in-charge declined to participate; and another because it was difficult to access the women after home birth during the wet season due to seasonal rivers and swamps.

Women: Within the antenatal clinics of participating health facilities, we included all pregnant women who were 28 weeks or more of gestation, and who had no plans to leave the district during pregnancy delivery or in the immediate postpartum period. We excluded women who had a planned elective caesarean-section delivery or previous caesarean section scars.

### Recruitment of women

Study staff briefed pregnant women attending the antenatal clinic about the study objectives and design of the study in a group. The key messages to the pregnant women included: 1. The benefits of delivering at the health facility, 2. Excessive bleeding after child-birth was dangerous to a woman’s life, 3. The availability of an effective drug (oxytocin) to stop excessive bleeding that can be given by trained health worker at the time of birth in the health facility. 4. For those willing to participate, the need to alert the research assistant by telephone when and where the delivery occurred. We repeated the sessions about the study in every antenatal clinic throughout the study period (in both control and intervention phases). After the discussion, we invited those eligible to participate. Each participant gave a written informed consent.

### Standard-of-care (control)

At the time of the trial, the standard-of-care was that a women who delivered at a health facility should receive oxytocin to prevent PPH, while women who delivered at home received no uterotonic.

### Intervention

Women in the intervention period were given 600 micrograms (mcg) of oral misoprostol at enrolment to the study to self-administer after childbirth if delivery happened outside a health facility, or when there was no oxytocin at the health facility. The three tablets of misoprostol (200 mcg each for a total of 600mcg) in aluminum foil were packaged in a plastic envelope.

### Instructions to women given misoprostol

Women were given the following instructions; “1. Not to take the misoprostol tablets when the baby is still inside the womb, 2. To swallow all the three tablets immediately after the birth of the baby, if delivery occurred at home, or if no oxytocin was given by the health provider. If she had twins, she was to swallow the tablets after the birth of second twin. 3. To keep the packaging of tablets (foil) after swallowing them and to give it to research assistant when she visits her. 4. To carry along the study tablets (misoprostol) when going to deliver at a health facility. Hand the misoprostol tablets to the attending midwife or research assistant if delivery occurred at health facility.”

### Training the study team

The research assistants and health facility staff in the antenatal clinics and delivery wards from the six study facilities were trained on the protocol for 5 h. This comprised of study material and key messages to women attending antenatal clinic, and was delivered by the principal investigator. Weekly supervisory visits by the principal investigator followed the initial training and further training was given as requested or as assessed by the principal investigator.

### Data collection

The study participants were interviewed face-to-face by a trained research assistant. We used a pre-tested questionnaire to collect socio-demographic characteristics including maternal age, education, marital status, maternal occupation and religious affiliation. We also inquired about parity, gestation at first antenatal visit, the use of prophylactic anti-malarials, transport costs to the health facility for antenatal care, and delivery plans. We established gestational age from the woman’s last normal menstrual period (LNMP) or ultrasound scan estimation. In a few cases where we did not have LNMP or an ultrasound scan, we used fundal height to approximate the gestational age [26]. Trained research assistants measured haemoglobin (Hb) levels at enrolment (during their third trimester antenatal care visit) and three to five days after delivery using a portable HemoCue^R^ Hb 301 system. as described in another part of the study that looked at haemoglobin status of pregnant women [27].

### Follow up of participants

All pregnant women enrolled in the study continued receiving standard antenatal care at the local health facility. A sticker identifying them as enrolled study participants was placed on their hand-held antenatal cards to make it easier to identify them at repeat antenatal visits or when they reported in labour. The sticker had three telephone numbers that the women could call to contact the study team once they had delivered or if they had any problems or questions about study. At enrolment, women were advised to deliver in a health facility as per national guidelines. They were also advised to seek care in case they had excessive bleeding after child birth, the placenta had not delivered within one hour, or the baby did not cry immediately after birth or developed a fever. The study kept a log of participants’ names, contact telephone numbers and the name of the village health worker where they lived. The study team contacted any woman who had passed her estimated delivery date to identify if she had given birth and from where. Midway through the study, we observed that the names of participants in the log book were often not what the women were called by the community members in the village, so we subsequently modified our procedures to ask participants for the names (petty names) the community members usually called them. Research assistants visited the woman either at home or at the health facility after birth to measure the haemoglobin and complete postnatal questionnaire. Participants were defined as lost-to-follow-up when we were unable to physically contact them eight weeks after the expected date of delivery.

### Outcome measures

The primary outcome was PPH, defined as a drop in maternal haemoglobin by 2g/dl or more, lower than the prenatal Hb [10].

Secondary outcomes were: postpartum anaemia defined as Hb < 11 g/dl when assessed within 7 days and Hb < 12 g/dl if assessed after the 7^th^ day after childbirth [28], place of child-birth, use of any uterotonics for prevention of PPH, referral to a health facility after delivery, blood transfusion and maternal death. We asked the women about side effects related to misoprostol use, such as fever (self-report of body feeling hot), chills, shivering and how they coped with them. Safety was defined as swallowing of the medicine after delivery of the baby or babies. Specific to the intervention group, we also assessed the timing of swallowing misoprostol, and its acceptability to women. We asked the women in the intervention group to keep the blister package of the misoprostol (used or unused) and hand it to research assistant at home during the follow up visit or to the nurse at the health facility where the woman delivered.

### Sample size

Sample size was calculated taking into account the clustering effect. We assumed a between cluster correlation coefficient k_m_ = 0.2, a minimum of 200 pregnant women per health facility in each phase (m), and proportion experiencing PPH of 12.0 % [9]. Assuming 80 % power to detect a difference of 50 % in PPH proportions between the two groups with a type I error of 5 %, using formula for matched cluster trial [29], the study needed six health facilities in each arm,

### Statistical analysis

Analysis was conducted on an intention-to-treat principle, based on the period (intervention or control) at which women were enrolled into the study. We compared the characteristics of women enrolled in the control and the intervention periods at individual level and these were summarized as percentages for categorical outcomes, and means (and standard deviations) for continuous outcomes. Cluster-level summaries of some women’s characteristics in the control and intervention periods were computed and presented as means and standard deviations.

The primary outcome (postpartum Hb ≥ 2g/dl lower than prenatal Hb) in each arm was expressed as the mean of the six cluster-level proportions of women who had PPH measured in each cluster in intervention and control periods respectively. The effect of the intervention was measured as the difference in the means of primary outcome of the two groups (with 95 % confidence interval [CI]). We used paired t-tests to assess whether the mean difference between the two groups was statistically different from zero. We also applied a paired *t*-test to assess the difference in the means of the secondary outcomes between the control and intervention groups. Uterotonic use included a summary statistic of uterotonic received at birth and was cross-tabulated with place of birth. The acceptability of misoprostol as a number of home births who ingested misoprostol and were willing to use it next pregnancy or recommend it to relative. Results were summarized as frequency distribution. Because the study did not have a lag phase, some of women recruited during the control period delivered in the intervention period.

## Results

### Participants flow

**Fig. 2:**
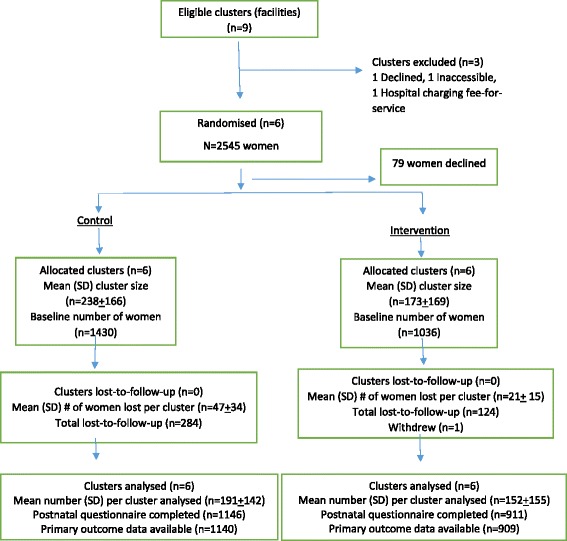
Participants flow diagram of the stepped-wedge, cluster randomized trial: All six facilities started as controls in the first step and two health facilities crossed over to the intervention arm in each step

All six clusters enrolled contributed to the control and intervention periods. Two thousand five hundred and forty-five women who came for antenatal care at the six health facilities during study period were eligible, and 2466 (97 %) consented to participate. A total of 409 women (17 %) were lost to follow up; 19.9 % in the control arm and 12.0 % in the intervention arm. Reasons for lost to follow up included being unable to contact women as phone contacts were switched off or change of address. Figure 2 shows the flow of study participants according to the Consort guidelines extension for cluster-randomized trials [30].

### Baseline characteristics

Table 1 shows the characteristics of the study participants enrolled in the control and the intervention periods. The baseline data established that randomization of the clusters to the two study periods were similar for almost all variables except for a lower prevalence of antenatal anaemia and HIV sero-status in women enrolled during the intervention period. The mean cluster size in control was more in control than in intervention period.

**Table 1:** Baseline characteristics of the study participants and clusters (*N =* 2466)

Variable	Control period *N =* 1430	Intervention period *N =* 1036
Individual level summary		
Age in completed years (mean, sd)	24.6 (±5.7)	24.2 (±5.5)
Below 20	261 (18.3 %)	221 (21.3 %)
20–24	548 (38.3 %)	400 (38.6 %)
25–29	409 (28.6 %)	278 (26.8 %)
30–34	101 (7.1 %)	76 (7.3 %)
≥ 35	111 (7.8 %)	61 (5.9 %)
Parity(mean, sd)	2.1 (2.0)	1.9 (1.9)
Nullipara	372 (26.0 %)	277 (26.7 %)
1	335 (23.4 %)	256 (24.7 %)
2	223 (15.6 %)	194 (18.7 %)
3	179 (12.5 %)	110 (10.6 %)
4	140 (9.8 %)	91 (8.8 %)
5+	181 (12.7 %)	108 (10.4 %)
Gestational age at enrolment (mean, sd)	32.4 (3.4)	32.1 (3.3)
Antenatal Hb in g/dl (mean, sd)	11.40 (1.44)	11.65 (1.28)
Antenatal anaemia (Hb<11.0 g/dl)^a^	512 (36.1 %)	279 (27.4 %)
HIV positive	122 (8.5 %)	71 (6.9 %)
Cluster level summary		
Cluster size (mean, sd)	238 (166)	173 (169)
Age in years (mean, sd)	24.5 (0.44)	24.5 (0.87)
Years at school (mean, sd)	7.6 (0.60)	7.5 (0.75)
Parity (mean, sd)	2.1 (0.23)	2.0 (0.44)
Antenatal anaemia (Hb<11.0 g/dl)^a^ (mean, sd)	36.7 (8.1)	30.7 (7.5)
HIV positive (mean, sd)	8.2 (2.0)	6.3 (1.4)

*Hb* Haemoglobin, *HIV* Human Immunodeficiency Virus, *sd* standard deviation

^a^Data on antenatal anaemia available for 1427 women in the control group and 1019 in the intervention group

Of the 2466 women recruited, 2057 (83.4 %) were successfully followed up. The median time to follow up was 17 days, ranging from 3 to 96 days postpartum. Among the women who were successfully followed up, the median (IQR) time from delivery to postpartum Hb measurement was 9 (4–22) and 7 (4–17) days in the control and intervention respectively. However 412/1140(36.3 %) in the control and 377/909 (41.4 %) in the intervention had the follow up done within 3–5 days.

One thousand seven hundred eighty-six (86.8 %) delivered at a health facility. One hundred fifty (7.3 %) women were delivered by caesarean section; 78/1146 (6.8 %) were in control arm while 72/911 (7.9 %) in intervention. Of the 271/2057 (13.2 %) women who delivered outside health facility, 168 (62.0 %) were assisted by traditional birth attendants, 141 (52 %) by relatives, 14 (5.2 %) were alone and 19 (7.0 %) by their husband. (*The sum is more than 271, some births were attended too by more than one person.*)

### Outcomes

#### Primary outcome

Overall, we measured maternal postpartum Hb in 2049/2057 (99.6 %) women who had a complete follow up done. Hb drop of ≥ 2g/dl was slightly more in control than in intervention period (18.5 % vs 11.4 % respectively; risk difference = 7.1%, 95 % CI -3.1 to 17.3, *p =* 0.14) Table 2.

**Table 2:** Main outcomes measures in control and intervention groups cluster level summaries

Outcome	Control group (*n*=6)	Intervention group (*n*=6)	Risk difference^c^ (Control-intervention) (95 % CI)	*p*-value
Primary outcome				
PPH Hb diff^a^ ≥2g/dl (sd)	18.5 % (5.9)	11.4 % (8.4)	7.1 % (-3.1 % to 17.3 %)	0.14
PPH Hb diff^b^ ≥2g/dl (sd)	15.0 % (4.3)	11.1 % (3.0)	3.9 % (-1.5 % to 9.3 %)	0.12
Secondary outcomes				
Postpartum anaemia (sd)	43.3 % (10.0)	41.3 % (11.0)	2.0 % (-2.4 % to 6.4 %)	0.30
Health facility births (sd)	87.5 % (7.3)	85.4 % (9.3)	2.1 % (-1.4 % to 5.6 %)	0.19
Uterotonic use at birth (sd)	80.4 % (12.6)	91.4 % (7.0)	−11.0 % (-25.7 % to 3.6 %)	0.11
Fever & shivering (sd)	14.9 % (3.4)	22.2 % (5.3)	−7.2 % (-11.1 % to -3.7 %)	0.005

*PPH* postpartum haemorrhage, ^a^Hb diff=postpartum haemoglobin - antenatal haemoglobin in women followed within 3^rd^ to 5^th^ day; n=412 in control and n=377 in intervention, ^b^Hb difference in all women; n=1140 in control and n=909 in intervention, ^c^ risk difference at cluster level are in means, *sd* standard deviation, *CI* confidence interval

#### Secondary outcomes

More women in the intervention period experienced fever and shivering (known side effects of misoprostol) compared to control period. However, there was no statistical differences between the intervention and control periods in any of the other secondary outcomes (postpartum anaemia, uterotonic use and facility births) Table 2. 5/893 (0.5 %) women in intervention period and 7/1118 (0.6 %) in the control period received blood transfusion.

Of the home deliveries (271/2057, 13 %), fewer women in control than intervention arm experienced Hb drop ≥ 2g/dl drop (9.6 % vs 14.5 % respectively), however, there was no statistical difference between the two arms (mean difference -4.5 %; 95 % CI -12.7 to 2.9, *p =* 0.17). Two women in each period were transferred from home to a health facility after child-birth for neonatal complications, none of which were related to misoprostol use.

##### Uterotonic use:

**Fig. 3:**
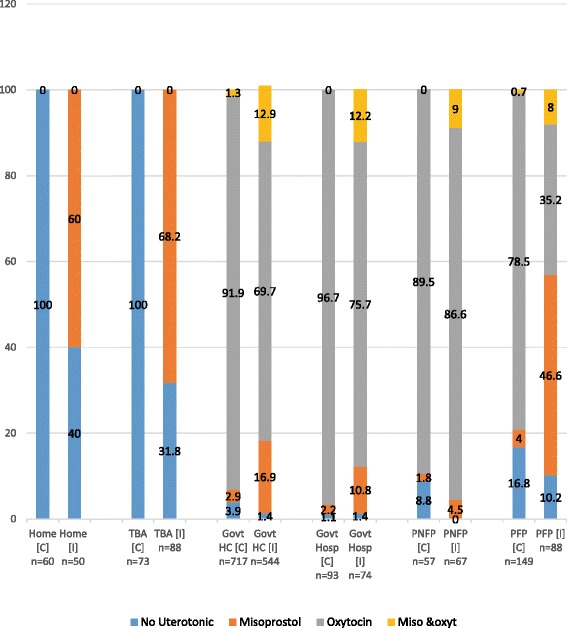
Participants’ use of uterotonics by place of delivery and study arm. C = control group, I = intervention group, TBA = traditional birth attendant, Govt HC = Government Health Centre, PNFP = private not-for-profit, PFP = private-for-profit

Figure 3 shows the women’s use of uterotonic by birth place and study group. Over 60 % women who gave birth outside health facility (home or traditional birth attendant’s home) in intervention arm self-administered misoprostol after birth. Among the women who delivered at a health facility, oxytocin was the main uterotonic used. However, when we considered only women who gave birth at the health facilities (public and private), receiving oxytocin was less common in intervention than in control period. This may be due to an unfortunate co-incidence of stock outs of oxytocin during the intervention period. Two hundred thirty-four women swallowed misoprostol despite delivering at a health facility and the following were reasons cited: facility stock-outs of injectable oxytocin (16.6 %), lack of syringes for administering oxytocin (15.7 %), told by the midwife to take the misoprostol (23.0 %), persistent bleeding despite receiving injectable uterotonic (so received both injectable and misoprostol) (16.6 %). However 24.7 % of women decided to take misoprostol without a justifiable reason. The private-for-profit health facilities had higher misoprostol use than other types of facilities. The misoprostol use in private-for-profit increased by 10 fold during the intervention period.

Among the 138 women who received misoprostol and delivered at home or in a traditional birth attendant’s home, 90 (65.2 %) used misoprostol (Fig. 3). Of the 48 women who did not swallow the misoprostol, 17 (35.4 %) of them forgot, 10 (20.8 %) misplaced it, 3 (6.3 %) did not have the pills at the time of delivery and 18 (37.5 %) decided not to take it.

### Acceptability of misoprostol

Of the 90 women who had home births and swallowed misoprostol, 85 (94.4 %) would use it in the next birth. The other five women opted not to have any more pregnancies. Eighty-seven (96.7 %) would recommend misoprostol to a friend or relative.

### Safety of misoprostol use

Of the 324 women who took misoprostol at birth (either in a health facility, at home or in a traditional birth attendant’s home), none took it before the baby was born (Table 3). However, the majority of women took the misoprostol after delivery of placenta, even though in the enrolment they were advised to take it after the baby is born but before the placenta was delivered. During study period, four maternal deaths were registered, and all of which occurred in control group. The causes of death were: sickle-cell crisis, PPH after caesarean section with postoperative haemorrhage, PPH secondary to abruption placenta, and alleged domestic violence.

**Table 3:** Timing of swallowing misoprostol tablet among the women who took it, by place of delivery

When misoprostol was swallowed	Home or TBA’s place *n*=90 (%)	Health facility *n*=234 (%)
Before baby was born	0	0
After the baby was born but before the placenta was delivered	32 (35.6 %)	74 (31.6 %)
After the placenta was delivered but within an hour of birth	58 (64.4 %)	159 (68.0 %)
More than 1 h after delivery	0	1 (0.4 %)

## Discussion

Antenatal distribution of misoprostol to women showed non-significant a reduction in the incidence of primary outcome of Hb drop ≥ 2g/dl at birth (7.1% [95 % CI -3.1 %, 17.3 %]). However it increased the use of uterotonic at birth more especially in private-for-profit health facilities and at home or at a traditional birth attendant’s home. Antenatal distribution also increased access to uterotonics at health facility births at times of oxytocin or syringe stock-out.

In this study, we found that more women delivered at a health facility (87 %) than reported in the National Demographic Health Survey 2011 (58 %) [21], however, there was no difference between the intervention and control group in the number of women presenting to deliver at the health facility. The women in the intervention reported more fever and shivering than women in control group (7.2 % [95 % CI 3.4 %, 11.1 %]), both common side effects of misoprostol. Majority of community births were assisted by traditional birth attendants and given the small number of women who delivered at home, there was no statistically significant difference between the groups in the rate of PPH.

Our study had the following limitations; it was not possible to mask the participants or the research team, to the intervention given its nature. Secondly, only 38.5 % of the women were seen within 5 days after birth, which affects the power of study and may introduce reporting bias of the primary outcome, however the mean difference in PPH between the two arms was not affected when all the women followed were included in analysis. Thirdly loss to follow-up of 17 % was high, however characteristics of women at enrollment were not different from those followed up (data not shown), and similar in the control and intervention groups. Different levels of loss-to-follow-up in the interventions (12 %) and control arms (20 %) may have been because we improved follow-up procedures by identifying petty names midway thought the study, when most clusters were intervention clusters. The strengths of the study include that the stepped-wedge design may increases the power of the study, since health facilities acted as their own control [22], and the study allowed us to assess the intervention in realistic setting.

The women in intervention group experienced lower PPH than the control group, however there was no statistical difference between the two groups. This is in contrast to community placebo randomized trials in India [9] and Pakistan [23] that showed a reduction of PPH in women who received misoprostol. The lack of observed reduction in PPH could have been due to the late follow up (more than 5 days) of majority of the participants that may cause inaccuracy in obtaining the true fall in Hb, knowing that women are subject to the natural rebound in the postpartum haemoglobin from day 7 after childbirth. The findings of no significant difference between the groups was possibly due to lower power of the study. The proportion of women with PPH among the intervention group was expected to be half lower than the control group according to the power calculations, but the results did not support reduction. The lack of observed reduction in PPH could also have been due to the a high rates of facility deliveries in both arms (possibly because this was emphasized during antenatal care, and in essence was one of the benefits observed arising from the study). This meant that many women got an injectable uterotonic, the standard of care in prevention of PPH. While this finding allays the concern of many policy makers who feared the antenatal distribution of misoprostol would encourage women to deliver at home, it made it harder to measure an effect. Our finding of more women returning to give birth at health facility supports population data from other studies reporting increased utilization of health facilities for deliveries in programmes implementing advanced distribution of misoprostol [31]. This means that the education and counseling to participants that went along with the distribution of misoprostol on the dangers of PPH, prompted many women to opt to deliver at the hands of skilled birth attendant or health facility in fear of complications of bleeding after childbirth.

Antenatally distributed misoprostol to women improved uterotonic use in institutional births. The increase in uterotonic utilization was noted more at private for profit health facilities. The use of misoprostol for PPH prevention in the private for profit health facilities increased from 4 % to 46 %. The possible explanation could be that misoprostol carried by the woman was an inducement for the staff at the private health facility not to use their stock of uterotonic, reducing the cost of items used at birth. In addition some of these private health facilities have stock outs of injectable uterotonic and misoprostol is cheaper for them and it does not require cold chain. Misoprostol used in addition to oxytocin in women who had institutional births and got persistent bleeding is opportunity for use of misoprostol for treatment of PPH.

Two-thirds (65.2 %) of the women who had home births and were in intervention group used misoprostol. This was encouraging, although it is less than the level of misoprostol use after home births reported in other settings (87.7 %–99 %) [13–15, 32–34]. The possible explanation for the slightly lower level of use in our study was that some women perceive bleeding after child birth as cleansing process and should not be inhibited. In addition, we gave misoprostol to some of the women as early as 28 weeks of pregnancy. There is increased chance to misplace the misoprostol or forget to use it. A recent review shows that early distribution of misoprostol in pregnancy is associated with lower rates of misoprostol use [11]. High levels of misoprostol use in home births is attributed to late pregnancy distribution (more than 32 weeks) [13, 14, 34] and use of village health workers as a distribution channel during home visits [13–15, 32]. So we may need to use the village health workers as distributors and see if there is a leap in number using misoprostol. Some studies register high rates because they use ancillary nurse midwives or TBAs to administer misoprostol at the time of birth [15, 34]. The eighteen women who decided not take misoprostol may have felt that they were not in danger. However, as earlier mentioned, some of these women may have considered bleeding after childbirth as a cleansing process and it is good for body. They perceive that stopping the bleeding process may cause the dirty things to stay in the body and is responsible for abdominal pains after childbirth, postpartum infection and later infertility. To them bleeding after childbirth should not be inhibited. The underlying belief that blood of childbirth is dirty has been reported among women in Morocco who also believe that blood of childbirth is bad and potentially poisonous inside the body [35].

Our study observed more women in the intervention phase than the control phase experienced fever and shivering, both of which are well known and documented side effects related to misoprostol use [7, 12, 36–38]. Fever and shivering were transient and the majority of women had symptoms subsiding within 2 h of misoprostol use. On the safety of antenatal distribution of misoprostol, our study had no incidence of misoprostol reported as being taken before the birth of the baby when it might harm the baby or lead to ruptured uterus, and all the women swallowed misoprostol after birth of the baby. This result concurs with reviewed literature that document very low rates (0.06 %) of mistimed administration of misoprostol [11] and this further allays concerns of the policy makers and international health community about the safety of antenatal distribution of misoprostol to women to self-administer in home births. However, a substantial proportion swallowed it after the placenta was delivered, which may not be within one minute after delivery of baby; a recommendation for active management of third stage of labour [39]. Similar findings by Smith et al and Weeks et al have showed that despite instructions of swallowing the tablets before birth of placenta, some swallow the tablets after placenta is delivered [14, 40]. The late administration of misoprostol may cause delay in onset of action and may ultimately contribute to the lack of effect on PPH. Though it is not well-established or known whether misoprostol administered after placental delivery significantly affects its uterotonic effect in reducing blood loss, we recommend a focused knowledge session and counseling at time the woman is offered misoprostol. In addition, a written pamphlet with instructions on how to swallow and the use of reminder messages given to women after distribution of the misoprostol either by phone “short text messages” or in subsequent visits would enhance remembrance of instructions on when to take the tablets.

Four maternal deaths that occurred during the study period, all of them from the control period and were not related to use of misoprostol or other uterotonics. The death of the woman who had PPH secondary to abruption placentae could have been averted if the health facility had capacity and skill to adequately treat a PPH. The facility had only intravenous fluids, oxytocin and misoprostol, which she received, but the inability of the health facility to carry out blood transfusion, or other temporizing measures like using balloon tamponade or anti-shock garments made the woman more vulnerable to death once she got a PPH [41, 42]. Acceptability of misoprostol was high with 94 % of women who gave birth at home and took misoprostol agreed or were willing to take it in the next pregnancy. They were ready to recommend it to a friend or relative.

## Conclusions

This study was unable to detect a significant reduction in Hb drops following the antenatal distribution of misoprostol. However, antenatal distribution of misoprostol to women, increased uterotonic use at birth and return to deliver at health facility. The safety of self-administration of misoprostol with close supervision and monitoring was demonstrated in this study. This study supports a potential strategy of antenatal distribution of misoprostol to women to self-administer for prevention of PPH, though more attention should be paid to educating women on when to take it in relation to delivery of the placenta.

## Additional file

Additional file 1: Checklist S1. CONSORT checklist for cluster randomised trials. (DOCX 27 kb)

## Abbreviations

DHO: District Health Office
Hb: Haemoglobin
HC: Health Centre
GOVT: Government
HIV: Human Immune-deficiency Virus
LNMP: Last Normal Menstrual Period
MDG: Millennium Development Goals
MCG: Micrograms
PFP: Private for profit
PNFP: Private Not for Profit
PPH: Postpartum Haemorrhage
TBA: Traditional Birth Attendants
WHO: World Health Organisation
